# The preclinical evaluation of immunocontraceptive vaccines based on canine zona pellucida 3 (cZP3) in a mouse model

**DOI:** 10.1186/s12958-018-0362-x

**Published:** 2018-05-11

**Authors:** Ying Wang, Yijie Li, Beibei Zhang, Fuchun Zhang

**Affiliations:** Xinjiang Key Laboratory of Biological Resources and Genetic Engineering, College of Life Science and Technology, Xinjiang University, 666, Shengli Road, Urumqi, 830046 China

**Keywords:** Dog, Zona pellucida 3, GnRH, Fusion protein, Contraceptive vaccine

## Abstract

**Background:**

Stray dogs are the reservoirs and carriers of rabies and are definitive hosts of echinococcosis. To control the overpopulation of stray dogs, zona pellucida 3 (ZP3), a primary receptor for sperm, is a potential antigen for developing contraceptive vaccines.

To enhance the immune responses and contraceptive effects of canine ZP3 (cZP3), dog gonadotropin-releasing hormone (GnRH) and a T cell epitope of chicken ovalbumin (OVA) were selected to construct two fusion proteins with cZP3, ovalbumin-GnRH-ZP3 (OGZ) and ovalbumin-ZP3 (OZ), and their contraceptive effects were evaluated in mice.

**Methods:**

The synthesized DNA sequences of *OGZ* and *OZ* were cloned into plasmid pET-28a respectively. The fusion proteins OGZ and OZ were identified by SDS-PAGE and Western blot. Mice were immunized with OGZ, OZ and cZP3, and the infertility rates were monitored. Mice immunized with mouse ZP3 (mZP3) or adjuvant alone were used as positive control and negative control, respectively. cZP3- and GnRH-specific antibodies (Abs) were detected by ELISA. The bindings of the Abs to oocytes were detected by indirect immunofluorescence assay. The paraffin sections of mice ovaries were observed under microscope for analyzing pathological characteristics.

**Results:**

SDS-PAGE and Western blot analyses showed that the two fusion proteins OGZ and OZ were correctly expressed. ELISA results showed that OGZ vaccine induced both cZP3- and GnRH-specific Abs, and OZ vaccine induced cZP3-specific Ab, which lasted for up to 168 days. The levels of follicle stimulating hormone (FSH) and estradiol (E2) in sera were significantly decreased in OGZ immunized mice. Indirect immunofluorescence results showed that Abs induced by cZP3 and mZP3 could bind to the mouse ZP and dog ZP each other. Compared with the adjuvant group, all vaccine immunized groups significantly decreased the fertility rate and mean litter size. Interestingly, the fertility rate in OGZ-immunized group is the lowest, and only 1 mouse out of 10 mice is fertile. Histological analysis of murine ovarian sections indicated that most of the infertile mice in the immunized groups lacked mature follicles as well as accompanied by inflammatory infiltration. Meanwhile, immunization with OGZ decreased the number of corpora lutea in the infertile mice.

**Conclusions:**

The fusion protein OGZ resulted in the lowest fertility rate and the least mean litter size in the immunized mice. OGZ might be a promising antigen for developing a new contraceptive vaccine for stray dog controlling.

## Background

Nowadays, the overpopulation of stray dogs has seriously affected peoples’ daily lives as well as city’s environmental sanitation. Stray dogs in Xinjiang, China, are the reservoirs and carriers of rabies, and they are definitive hosts of echinococcosis. Therefore, it is urgent to develop effective contraceptive vaccines to control stray dog’s population. Zona pellucida 3 (ZP3), as the primary receptor of sperm on egg cell and an inducer of the acrosome reaction, plays a key role during fertilization [1–4]. Extensive studies have demonstrated that contraceptive vaccines based on ZP3 can limit the population of mice [5–7], koalas [8], gray kangaroos [9], and rabbits [10]. Recombinant canine ZP3 (cZP3) can also induce infertility in female dogs [11]. In this study, cZP3 of 35~350aa containing the major B-cell epitopes was selected as the basic antigen for immunocontraception.

To enhance the contraceptive efficacy of cZP3, canine gonadotropin-releasing hormone (GnRH) was selected as another antigen. GnRH plays an important role in vertebrate fertilization. It is a decapeptide produced by hypothalamus. Immunization with GnRH can produce contraceptive effects in both males and females [12–14]. Several commercial GnRH-based contraceptive vaccines have been developed, and these vaccines appear to have different functions in different animal species [15–17]. Thus, canine GnRH was selected as the second antigen for preparing a fusion protein vaccine. In addition, a T-cell epitope (QAVHAAHAEINE) of chicken ovalbumin (OVA) was added to the N -terminal of the fusion protein to further enhance the immune responses [18].

In this study, we chose cZP3 as the basic antigen for constructing two fusion proteins that encompassed a T cell epitope of OVA and, or GnRH. The contraceptive efficacy of the two fusion proteins were evaluated in female mice. The related Ab levels and the binding of Abs on ZP3 of oocytes were conducted to explain the mechanisms underlying the contraceptive effect.

## Methods

### Animals

All animal experiments in this study were approved by the Animal Ethics Committee of Xinjiang University. Treatment and care of animals were conducted strictly according to the guidelines, and all efforts were made to minimize damages to the animals. All mice were maintained under constant room temperature (21 ± 2 °C) with a photoperiod of D 12:12. Mice had free access to food and water. No mice died during the experiments.

### Construction, expression and purification of the fusion proteins

**Fig. 1:**
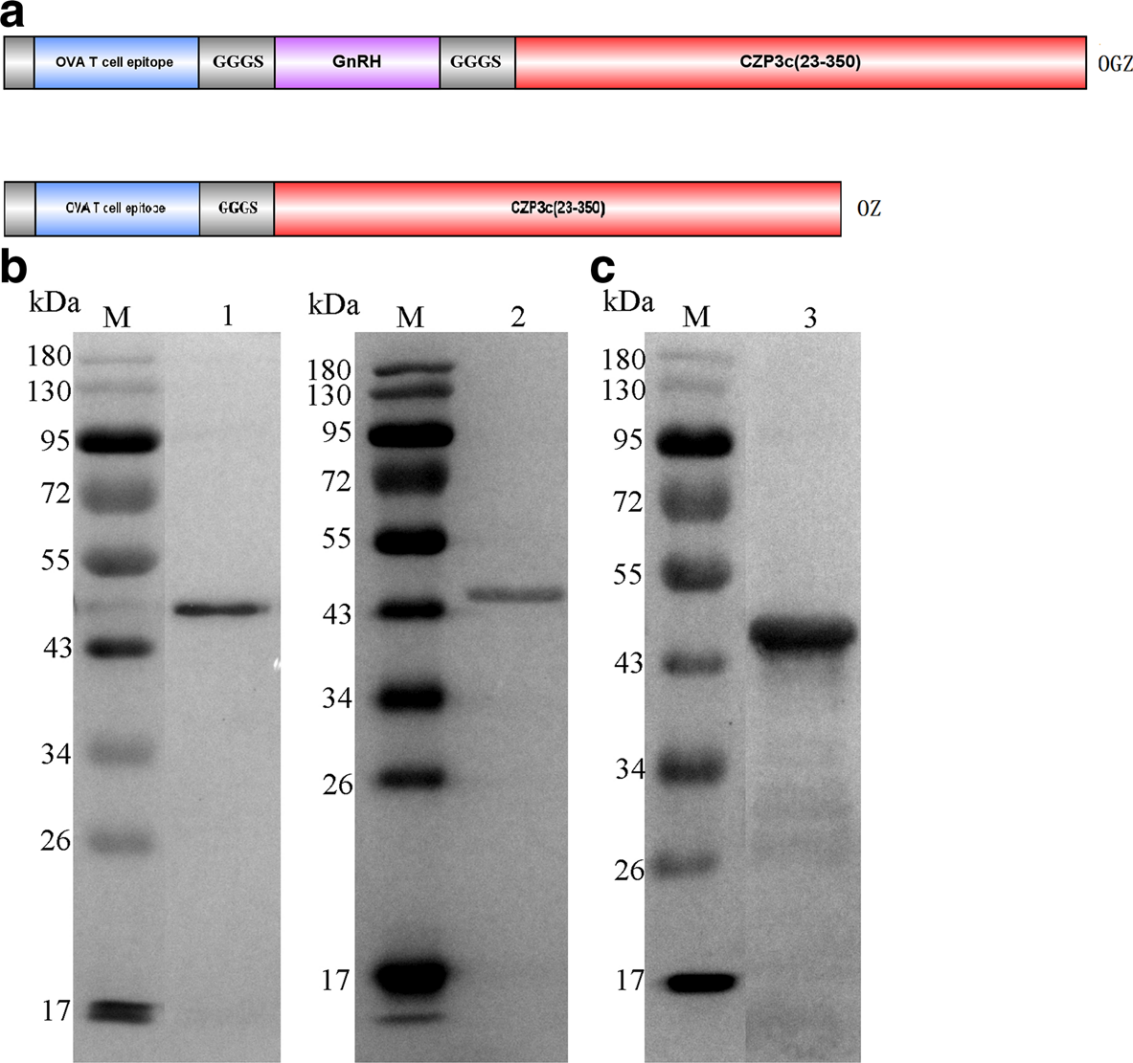
Schematic representation and Western blot analysis of the fusion proteins OGZ and OZ. **a**. The main components of OGZ were T cell epitope of OVA, GnRH and cZP3. The main components of OZ were T cell epitope of OVA and cZP3. A flexible linker (GGGS) was inserted in between the components. **b**. OGZ and OZ reacted with antibodies (Abs) against cPZ3. Lane 1: OGZ, lane 2: OZ. **C**. OGZ reacted with Abs against GnRH. Lane 3: OGZ. Lane M: protein molecular weight markers

Two recombinant fragments *OGZ* and *OZ* were constructed. For *OGZ*, canine GnRH (G) and cZP3 (23~ 350 aa) (Z) nucleotide sequences were retrieved from GenBank (XP_850859.2 and NM 001003224.1). A T-cell epitope of chicken ovalbumin (O) was added to the 5′-end of the recombinant fragment. A flexible linker (Gly-Gly-Gly-Gly-Ser) (GGGS) was inserted to separate each component in the fusion protein (Fig. 1a). *OZ* recombinant fragment was constructed same as OGZ except for missing the GnRH and the second GGGS (Fig. 1a). The synthesized DNA sequences were codon-optimized for *E. coli* expression, and the expressed fusion proteins were named as OGZ and OZ, respectively. The nucleic acid fragments of *OGZ* and *OZ*, were digested with *Eco*R I and *Bam*H I, and cloned into pET28a vector respectively. The recombinant plasmids pET28a-OGZ and pET28a*-*OZ were transformed into *E. coli* BL21 (DE3) cells respectively. The expressional conditions were optimized by testing different combination of factors, including isopropyl β-D-1-thiogalactopyranoside (IPTG) concentration (0.1, 0.3, 0.5, 0.8, 1, 1.5 and 2 mmol/L), induction time (4 h, 6 h and 8 h) and temperature (25 °C, 30 °C and 37 °C). Finally, the optimal induction conditions in 1 L LB medium for OGZ or OZ expression was as follows: 10 mL overnight culture was inoculated into 1 L LB medium and cultured at 37 °C with shaking at 250 r/min. When OD_600_ reached between 0.4~ 0.6, 1 mM IPTG was added into the culture. The culture was continually incubated at 37 °C for 4 h. Cell pellets were harvested by centrifugation at 5000 r/min for 15 min and washed once with phosphate buffered saline (PBS). Then, the pellets were resuspended in PBS (100 mg/2 mL, containing 20 μL protease inhibitor cocktail) and sonicated in an ice bath for 20 min at 5 s intervals. The cell lysate was harvested by centrifugation at 10000 r/min for 15 min, and the pellets were resuspended in 20 mL binding buffer (20 mM Tris-HCl, pH 7.9, containing 6 M urea, 0.5 M NaCl, and 5 mM imidazole). After spinning at 10000 r/min for 20 min, the filtrated supernatant was passed through a nickel-affinity chromatography column three times and washed 20 times with a column volume of binding buffer. The fusion proteins were eluted by elution buffer (500 mM imidazole). After ultrafiltration, protein concentrations were determined using a BCA Protein Assay Kit (Thermo).

### Western blot analysis of the fusion proteins OGZ and OZ

The antisera against cZP3 (23~ 350 aa) and GnRH were raised respectively by one subcutaneous and two intraperitoneal injections in female mice.

The transformed *E. coli* cells were induced with IPTG to express OGZ and OZ respectively. The whole cells were collected by centrifugation at 10000 r/min for 1 min and washed once with PBS. The pellets were dissolved in SDS-PAGE loading buffer and heated at 100 °C for 5 min. The samples were then separated on a 12% SDS-PAGE gel and transferred onto PVDF membranes separately. The membranes were blocked with 5% skim milk powder dissolved in 0.5% Tween-20 in TBS pH 7.9 (TBST) at 4 °C overnight. The membranes were then probed with different anti-sera. For OGZ and OZ, the membranes were probed with Abs against cZP3. OGZ was also probed with Abs against GnRH. After incubation at 4 °C for 2 h, the membranes were washed with TBST three times. Horseradish (HRP)-conjugated goat anti-mouse IgG was used as a second Ab. Color was developed with 3, 3′-diaminobenzidine (DAB). All reactions were terminated by adding distilled water.

### Mouse immunization

Fifty female BALB/c mice (6~ 8 weeks old) were purchased from Xinjiang Medical University and were randomly divided into five groups (*n* = 10). Immunization with OGZ, OZ and cZP3 (23~ 350 aa) was designated as the antigen-specific groups. Immunization with mouse ZP3(mZP3, 21~ 361 aa) was designated as positive control. cZP3 and mZP3 proteins were previously expressed and purified in our laboratory. Freund’s adjuvant injection was set as a negative control.

After the protein was emulsified with Freund’s complete adjuvant, each mouse received 25 μg protein subcutaneously. The same amount of protein was given intraperitoneally as a booster dose on day 21 and 42 after administration of the first immunization. Serum samples were collected from the retro-orbital on day 14, 35, 56, and 168 after the first immunization. 70 μL sera were collected from each mouse, and were stored at − 80 °C for later use.

### Detection of the abs levels by enzyme linked immunosorbent assay (ELISA)

We detected the Ab levels of ZP3 and GnRH using ELISA. A ninety-six-well plate was coated with 100 μL ZP3 (4 μg/mL) and incubated at 4 °C overnight. The plate was blocked with blocking buffer (5% skim milk powder in PBST) at 37 °C for 1 h. After washing three times, the plate was incubated with serum (100 μL per well) diluted at 1:1000 in blocking buffer at 37 °C for 1 h. Then, the plate was incubated with the second Ab, HRP-conjugated goat anti-mouse IgG at a dilution of 1:1000, at 37 °C for 1 h. TMB substrate solution (Thermo) (50 μl per well) was used for color development. After 15 min, the reaction was terminated by adding the same volume of 0.2 M H_2_SO_4_. Absorbance values were read at OD_450_ nm.

The GnRH Ab levels were detected as described above. The optimal coating concentration of GnRH was 15 μg/mL and the working concentration of the serum samples was 1:100 dilution as determined by chessboard assay.

### Determination of the levels of FSH and E2 in the sera of OGZ immunized mice by ELISA

The levels of FSH and E2 in the mice sera of adjuvant and OGZ groups were determined respectively by sandwich and competitive ELISA according to the manufacturer’s instructions (Elabscience, Wuhan, China). Serum samples were collected on day 14, 35 and 56 after the first immunization. The assay sensitivity was 1.56~ 100 ng/mL and 40~ 1400 pg/mL, respectively. The intra- and inter-assay of the variations for FSH and E2 was 10 and 15%, respectively.

### Indirect immunofluorescence of mouse oocytes

Five 6-week-old female BALB/c mice were stimulated by intramuscular injection with 12 IU of pregnant mare serum gonadotrophin (PMSG, Ningbo Sansheng Pharamceutical, China). 46 h later, these mice were injected intramuscularly with 12 IU human chorionic gonadotropin (hCG, Ningbo SanSheng Pharamecutical, China). All the mice were sacrificed after 13 h of the injection, and the cumulus-oocyte complexes (COCs) were collected from the mice ampulla. After incubation with hyaluronidase (50 μg/mL) for 5 min, the denuded oocytes were obtained. The oocytes were blocked with blocking buffer (5% BSA in PBS) at 37 °C for 1 h, and then were transferred into the freshly diluted serum droplets (1:50) for 1 h. After the oocytes were washed several times with PBS, fluorescein isothiocyanate (FITC)-conjugated rabbit anti mouse IgG (1:200) was added, then the oocytes were incubated at 37 °C for 30 min. Finally, the oocytes were washed with PBS and observed under fluorescence microscope.

### Indirect immunofluorescence of dog ovarian sections

For detecting whether the antisera of the OGZ- and OZ- immunized mice could bind to dog oocytes, we performed surgery and collected four ovaries from two Beagles. The ovaries were immediately fixed in 4% paraformaldehyde and kept at 4 °C for at least 24 h, and then were made into paraffin sections. The sections were blocked with PBS containing 10% normal rabbit sera at 37 °C for 1 h. Serum samples (from the mice of adjuvant, OGZ, OZ, mZP3 and cZP3 injection) were diluted with blocking buffer (1:50), and were added onto the sections, respectively, and kept at 4 °C overnight. The diluted FITC conjugated rabbit anti mouse IgG (1:200 in blocking buffer) was added as a second Ab. After washing, the paraffin sections were observed under fluorescence microscope.

### Evaluation of the contraceptive effects in vivo

On day 56 after the primary immunization, mice in each group were divided into five cages (two mice per cage) and a healthy male mouse was put into each cage. The male mice were rotated in the cages every day. After 3 weeks, the male mouse was removed from the cage, and the litter size of each female mouse was counted. On day 168, the mating test was repeated.

### Histological analysis

On day 210 after the first immunization, all of the mice were sacrificed. The ovaries from each mouse was obtained and fixed in 4% paraformaldehyde at 4 °C. After embedded with paraffin, the ovaries were cut into two consecutive sections at 5 μm thickness. The sections were stained with hematoxylin and eosin, and observed under microscope. Histopathological changes of the ovaries were graded 0 to 4 as previously described [19].

### Statistical analysis

The Abs levels for FSH, E2 and GnRH were analyzed by one-way analysis of variance (one-way ANOVA) and Tukey’s multiple comparison test. The Ab levels of ZP3 during the immunization were analyzed by two-way ANOVA. The correlation between the sera FSH concentration and the Ab levels of GnRH was analyzed using Pearson’s correlarion coefficient. For analysis of the difference in mean litter size among each group, the data was firstly converted into square root of *x* + 1, then analyzed by one-way ANOVA and Tukey’s multiple comparison test. The Ab levels and the mean litter size were presented as mean ± SEM. *P* <0.05 was considered as significant.

## Results

### Purification and detection of the fusion proteins OGZ and OZ

Fusion protein OGZ was combined with canine GnRH (G), cZP3 (Z, 23~ 350 aa) and a T-cell epitope of chicken ovalbumin (O). Fusion protein OZ was combined with cZP3 (Z, 23~ 350 aa) and chicken OVA (O) (Fig. 1a). After these two proteins were expressed in *E.coli* by IPTG induction, and after proteins purification, the fusion protein OGZ and OZ showed a major band at 46 kDa and 44 kDa on SDS-PAGE gel respectively as expected (figure not shown). Western blot results confirmed these specific bands accordingly. Both OGZ and OZ could bind to the Abs against cZP3 (Fig. 1b). Besides, OGZ also bound to Abs against GnRH (Fig. 1c).

### Fusion proteins induced high levels of abs against ZP3

**Fig. 2:**
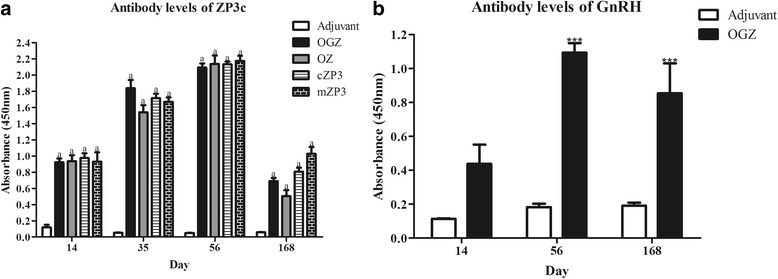
Antibody levels of ZP3 and GnRH in serum. Sera were collected from the mice on day 0, 14, 35, 56 and 168 after the 1st immunization. **a.** Ab levels of ZP3. All the serum samples were diluted (1:1000) with PBST containing 5% skim milk. HRP-conjugated goat anti-mouse IgG Ab (1:1000) was used as the second Ab. **b.** Ab levels of GnRH. Serum samples were collected from the mice on day 14, 56 and 168 at 1:100 dilution. Data are shown as mean ± SEM. ***P<0.001

From day 14 to day 56 after the first immunization the levels of the Ab against ZP3 in all proteins immunized mice were significantly higher than the adjuvant control group. The increase of the Ab levels was in a linear way with the time prolonging. On day 168 the Ab levels decreased greatly but still kept 5~ 10 fold of the adjuvant control (Fig. 2a). There was no significant difference among these protein groups. In addition, OGZ group could also generate high levels of Ab against GnRH and the levels also last for 168 days (Fig. 2b). However, the levels of Ab against GnRH were not as high as that of ZP3.

### Sera FSH and E2 levels were significantly decreased in OGZ group

**Fig. 3:**
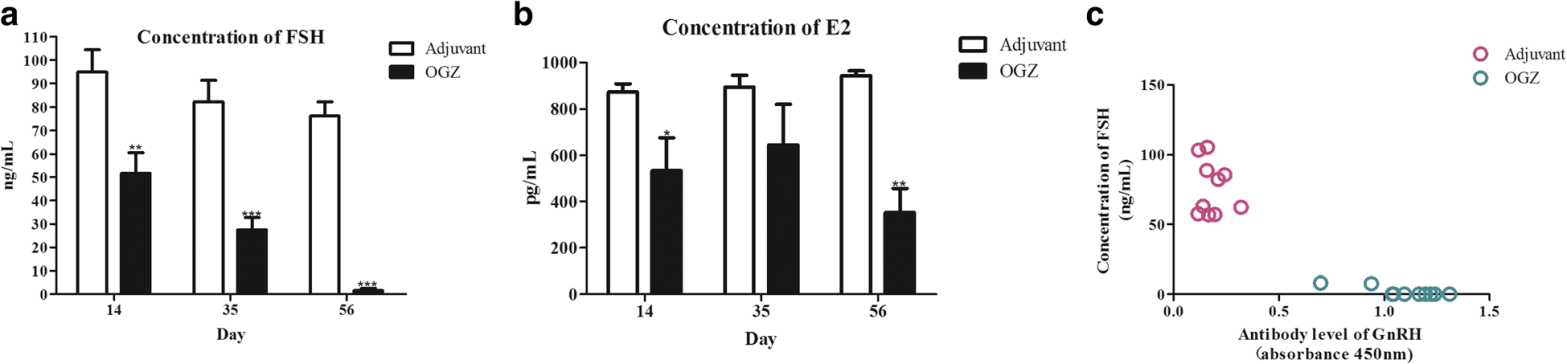
Concentration of FSH and E2 in sera in adjuvant and OGZ groups. **a.** FSH concentration in sera. **b.** E2 concentration in sera. Serum samples collected on day 14, 35 and 56 post the 1st immunization were detected by ELISA Kits. Data are shown as mean ± SEM. *** indicates *P*<0.001, ** indicates *P*<0.01, and * indicates *P*<0.05. **c.** Correlation between Ab levels of GnRH and the concentration of FSH. There is a negative correlation between Abs levels of GnRH and the concentration of FSH in OGZ groups. Pearson’s correlation coefficient *r* value is − 0.6403, *P*<0.05

The sera FSH and E2 levels in the OGZ group showed a gradual decline (Fig. 3a, b) after immunization, and for FSH it was almost undetectable on day 56 after the first immunization. The FSH concentration in the sera of OGZ group was negatively correlated with the Ab levels of GnRH (Fig. 3c) with a Pearson’s correlation coefficient of − 0.6403.

### Abs against fusion proteins could bind to the ZP matrix of mouse and dog

**Fig. 4:**
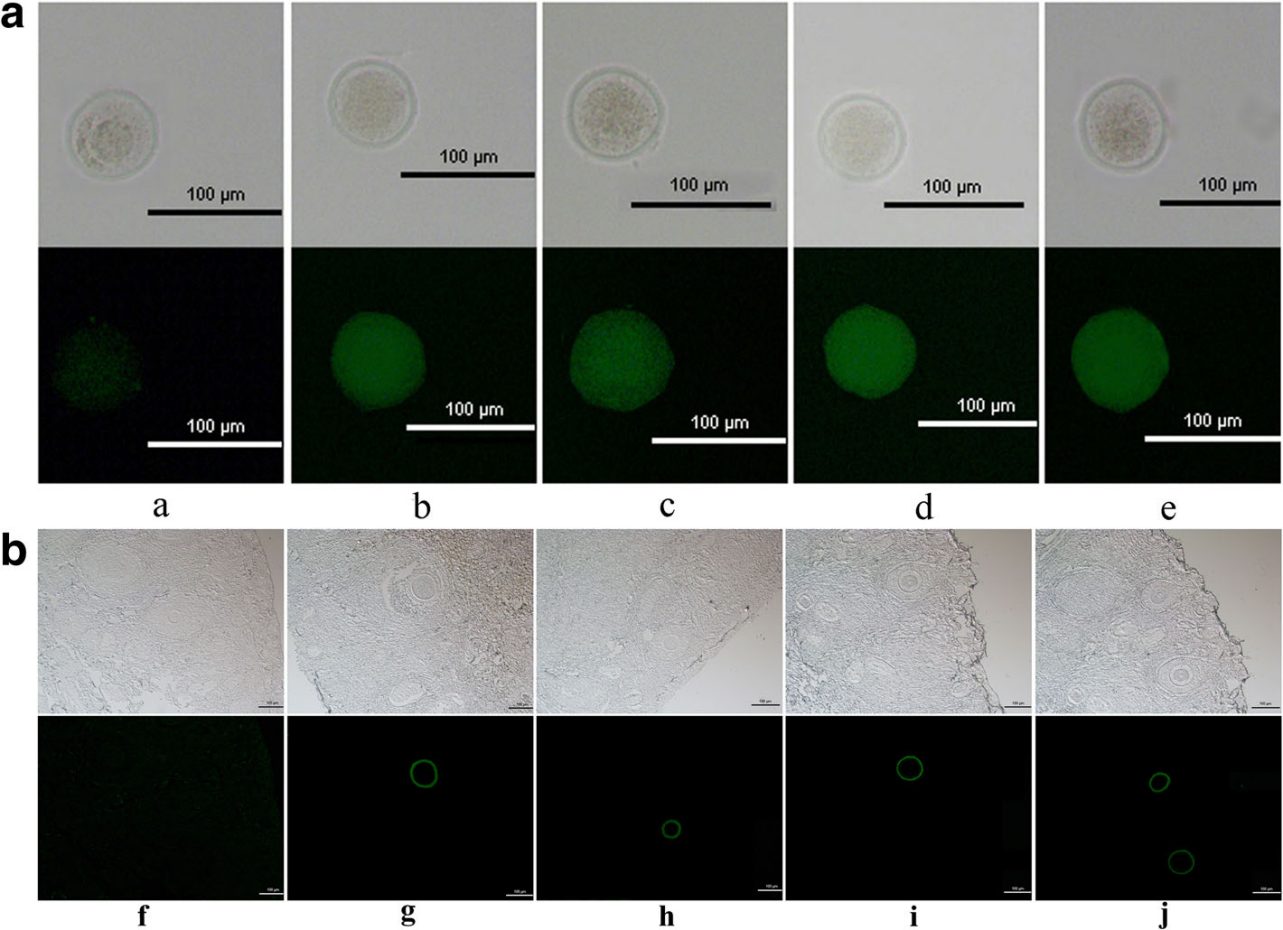
Abs against ZP3 could react with mouse and dog ZP in the indirect immnofluorescence assay. **a**. Abs against ZP3 react with mouse oocytes. **a**, **b**, **c**, **d** and **e** represent adjuvant, OGZ, OZ, cZP3 and mZP3, respectively. **b**. Abs against ZP3 reacted with sections of dog ovaries. **f**, **g**, **h**, **i** and **j** represent the adjuvant, OGZ, OZ, cZP3 and mZP3, respectively. The top panel is in bright field, the bottom panel is in fluorescent field

Indirect immunofluorescence assay of mice oocytes showed that the Abs against ZP3 in each vaccine immunization group could specifically bind to the ZP matrix of mice (Fig. 4a). Meanwhile the results of indirect immunofluorescence assay to dog ovarian sections suggested the Abs against ZP3 in each vaccine group could react with the dog ZP matrix, but not with other cells in ovaries (Fig. 4b).

### Immunization with OGZ and OZ both caused a decline in mice fertility

To detect the contraceptive effects of the fusion proteins, the OGZ and OZ immunized female mice were mated respectively with male mice for 3 weeks on day 56 after the first immunization. The results showed that both the fertility rate and litter size in OGZ group and OZ group decreased significantly compared to the adjuvant group, so did the mZP3 and cZP3 groups. The fertility rate decreased from 100% in the adjuvant group to 10% in OGZ group, 40% in mZP3 group and 50% in cZP3 and OZ groups (Table 1).

**Table 1:** Contraceptive effects of the recombinant proteins in the first mating test

Antigen	Fertility rate (%)^a^	Mean litter size^b^	Average Abs levels	*P* ^c^
Adjuvant	100% (10/10)	7 ± 0.516	0.050 ± 0.004^d^	
			0.183 ± 0.02^e^	
OGZ	10% (1/10)	0.4 ± 0.4	2.092 ±0 .162 ^d^	***
			1.095 ± 0.056 ^e^	
OZ	50% (5/10)	2.3 ± 0.883	2.138 ±0 .334 ^d^	**
cZP3	50% (5/10)	2.2 ± 0.772	2.134 ±0 .103 ^d^	**
mZP3	40% (4/10)	2 ± 0.907	2.215 ±0 .156 ^d^	***

^a^Fertility rate: number of the fertile mice/total number of the mated mice in each group

^b^Mean litter size of mice: total number of pups in each group/total number of the mated mice in each group

^c^Mean litter size in each group vs adjuvant group;* represents *P* < 0.05; ** represents *P* < 0.01; *** represents *P* < 0.005

^d^Average Abs levels of cZP3

^e^Average Abs levels of GnRH

To detect how long the contraceptive efficacy of OGZ and OZ vaccines could last, the mating test was re-conducted on day 168. The results showed that the fertility rate and litter size in each group increased slightly compared to the first mating, but still kept a significant decrease compared to the adjuvant group. The fertility rate decreased from 100% in the adjuvant group to 10% in the OGZ group and 50% in OZ, cZP3 and mZP3 groups (Table 2).

**Table 2:** Contraceptive effects of the recombinant proteins in the second mating test

Antigen	Fertility rate (%) ^a^	Mean litter size ^b^	Average Abs levels	*P* ^c^
Adjuvant	100% (10/10)	6.1 ± 0.924	0.058 ± 0.003 ^d^	
			0.191 ± 0.018 ^e^	
OGZ	10% (1/10)	0.2 ± 0.2	0.691 ± 0.039 ^d^	***
			0.854 ± 0.176 ^e^	
OZ	50% (5/10)	2.5 ± 0.86	0.506 ± 0.073 ^d^	*
cZP3	50% (5/10)	2.6 ± 0.884	0.808 ± 0.047 ^d^	*
mZP3	50% (5/10)	2.7 ± 0.920	1.028 ± 0.084 ^d^	*

^a^Fertility rate: number of the fertile mice/total number of the mated mice in each group

^b^Mean litter size of mice: total number of the pups in each group/total number of the mated mice in each group

^c^Mean litter size in each group vs adjuvant group;* represents *P* < 0.05; ** represents *P* < 0.01;*** represents *P* < 0.005

^d^Average Abs levels of cZP3

^e^Average Abs levels of GnRH

The average litter size in OZ group was 2.3, similar to the groups of mZP3 which was 2 and cZP3 which was 2.2, and all of them were very significantly lower than the adjuvant group which was 7 (Table 1). The OGZ group had the least average litter size of 0.4 (out of 10 mice only 1 produced 4 pubs). The results in the second mating test showed similar effects (Table 2). These results suggested that these protein vaccines effectively decreased the litter size of the immunized mice, and OZ, cZP3 and mZP3 had similar moderate effect, while OGZ had very strong effect.

**Fig. 5:**
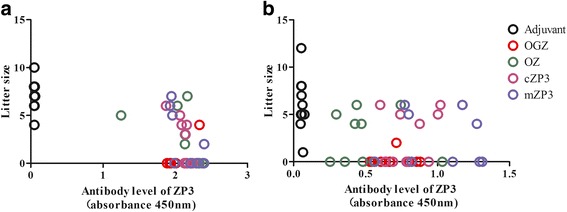
Scatter diagram of the antibody levels of cZP3 in each mouse and the litter size of each mouse after the first mating (**a**) and the second mating (**b**)

The scatter diagrams about the Ab levels of ZP3 and the litter size in each mouse (Fig. 5) showed that all of the mice in the protein groups had higher Ab levels than the adjuvant control, and the litter size was smaller than the adjuvant (Table 1 and Table 2). However, the difference in the litter size among the individuals in each group was great, for OZ, cZP3 and mZP3 groups, the range were 6, suggesting that random factors had influence on a mouse’s litter size apart from the main factor of the contraceptive vaccine. This situation was even evidence in the adjuvant group (Fig. 5), the range was 11. Due to the data severely deviated from normal distribution, Pearson’s correlation analysis were not applicable.

### Immunization with OGZ and OZ led to pathological changes in mice ovaries

**Fig. 6:**
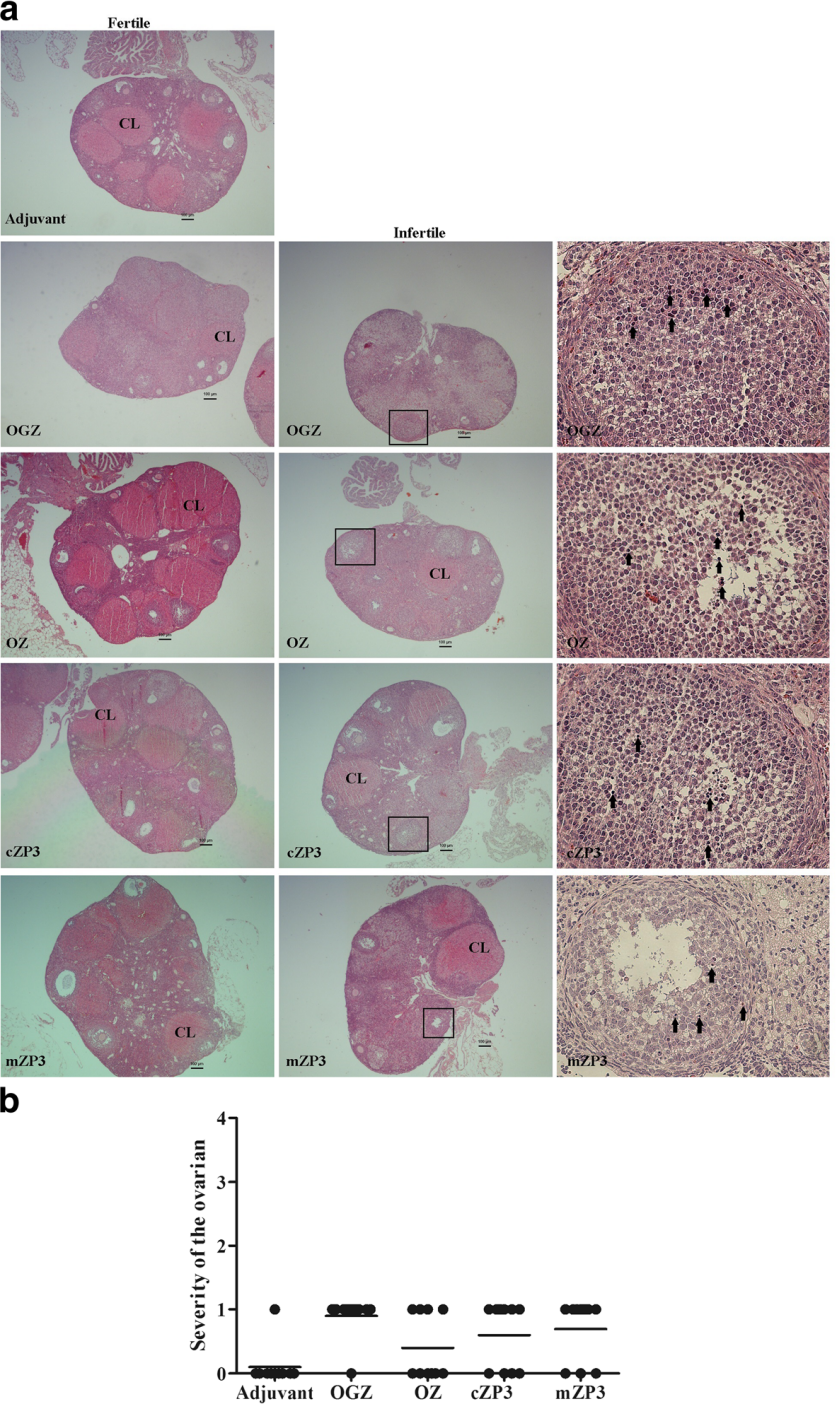
Histological analysis of the ovaries of the mice immunized with different ZP3 vaccines. Mice ovaries were collected on day 210 after the first immunization. The paraffin sections were observed after HE staining. **a**. Histological analysis of the ovaries of the fertile and infertile mice in each group. The magnified(200×)image of the black box are on the right. Black arrows indicate inflammatory cell infiltration. CL represents corpus luteum. **b**. Severity of the ovarian lesions in different immunized groups

To investigate whether immunization with the fusion proteins could affect mice ovarian morphology, the mice ovaries were collected, and sections were made for observation. We found that, compared with the adjuvant group, most of the infertile mice immunized with protein vaccines had abnormal follicular development, which led to immature follicles. In additon, these ovaries were also accompanied by inflammatory cell infiltration in the atretic follicles and/or the growing and mature follicles. The number of corpora lutea in the infertile mice receiving OGZ also decreased. Most of the fertile mice in these protein immunization groups showed no significant changes in the number of corpora lutea (Fig. 6).

## Discussion

In face of the overpopulation of stray dogs, developing a safe and effective contraceptive vaccine is in need. ZP3, due to the vital function in the fertilization process, is considered as a promising target for such a vaccine. In order to induce a stronger immune response, two recombinant fragments *OGZ* and *OZ* were constructed. The fusion proteins OGZ and OZ were correctly expressed in *E.coli*.

Both of the mature fragment of mZP3 and cZP3 induced strong immune responses in female mice and lead to infertility as expected. OGZ and OZ also induced strong immune responses, there were no significant differences in the anti-ZP3 Ab levels among the protein immunization groups. Compared to cZP3, mZP3 encompassed all the epitopes on mice ZP matrix, however, it did not elicit the highest Ab level as predicted (Fig. 2a). This is probably because cZP3 has stronger immunogenicity than mZP3 to mouse and stimulated stronger immune responses [20]. This result suggested that in developing contraceptive vaccines for controlling mammalians heterologous ZP3 might be more prospective. Additionally, the anti-GnRH Ab level was lower than that of anti-ZP3 (Fig. 2b), and this was probably due to the differences in the coating antigen. The Ab levels detected by ELISA with fusion proteins as coating antigens are usually higher than synthetic peptides as coating antigens, and this difference is similar to the results of previous studies [21, 22].

The protein vaccines showed significant contraceptive effects on the immunized female mice. Both the fertility rate and litter size decreased significantly in comparing with the adjuvant group. The effect of OGZ was profound, in the first mating test, 9 out of the 10 mice gave no birth, the only fertilized one only produced 4 pubs which was lower than the average litter size which was 2~ 2.3 in the other ZP3 protein groups. These results suggested that OGZ vaccine not only blocked ZP3 but also blocked GnRH, thus led to the highest infertility rate (9/10) than OZ(5/10), cZP3(5/10) and mZP3(4/10).

The Abs against GnRH bind to GnRH to prevent GnRH from interacting with the receptors, which, in turn, disturb the hypothalamic-pituitray-gonad axis and inhibit downstream hormonal activities, such as the secretion of FSH and E2 [23, 24]. FSH stimulates the production of E2, and a decrease in FSH concentration could suppress the synthesis of E2. In this study, Abs against GnRH in the female mice were adequate for interacting with GnRH and resulting in the decrease of E2 concentration. Compared to adjuvant, the sera FSH and E2 levels in OGZ group were remarkably reduced (Fig. 3a, b). These results were similar to other studies in which immunization with GnRH caused a significant reduction in FSH and E2 concentration [21–27]. Lower concentration of FSH and E2 possibly resulted in ovary dysfunction and infertility.

The results of the indirect immunofluorescence assay showed that the Abs against cZP3 had the affinity to ZP matrix of mice and dogs. This may be attribute to the sequence similarity of cZP3 and mZP3, they shared 65% identity [28].

A large body of evidence indicates that ZP3 induces infertility via two mechanisms: (1) sufficient anti-ZP3 Abs bind to ZP matrix and block the sperm-egg interactions; (2) ovarian dysfunction mediated by inflammatory cell infiltration and anti-ZP3 Abs [29–34]. In this study, most of the infertile mice showed aberrant development of ovarian follicles, which had inflammatory cell infiltration and lack of mature follicles (Fig. 6a). The normal ovarian functions were interfered with Abs against ZP3 and/or inflammatory cells, which might lead to infertility. Our results agreed with this explanation.

Other studies report that ovaries receiving GnRH present a decrease in the number of corpora lutea in the infertile mice, and that lead to follicular dysplasia [24, 35–37]. Our results in OGZ group were in accord with those studies. The decrease in the number of corpora lutea and mature follicles was consistent with the decreases in sera FSH and E2 concentrations in the OGZ immunization group. These findings indicate that the Abs against cZP3 and GnRH worked together to lead to serious infertility with only 10% fertility rate and an average of 0.4 litter size in the OGZ group.

## Conclusions

The results in this study showed that fusion protein OGZ had very stronger effect on reducing the fertility rate and litter size of the immunized mice than cZP3 and mZP3 by inducing high Abs levels of anti-ZP3 Abs and anti-GnRH Abs which not only blocked ZP3 but also blocked GnRH. OGZ immunization also caused pathological changes in ovary, which led to ovarian dysfunction and further increased infertility. Thus, the fusion protein OGZ could be a promising candidate for developing contraceptive vaccines for stray dogs controlling.
